# Structure and Corrosion Behavior of Multiphase Intermetallic ZrCu-Based Alloys

**DOI:** 10.3390/ma17174182

**Published:** 2024-08-23

**Authors:** Rafał Babilas, Katarzyna Młynarek-Żak, Aneta Kania, Akash A. Deshmukh, Tymon Warski, Łukasz Hawełek

**Affiliations:** 1 Department of Engineering Materials and Biomaterials, Silesian University of Technology, Konarskiego 18a, 44-100 Gliwice, Poland; aneta.kania@polsl.pl; 2 Department of Engineering Processes Automation and Integrated Manufacturing Systems, Silesian University of Technology, Konarskiego 18a, 44-100 Gliwice, Poland; katarzyna.mlynarek-zak@polsl.pl; 3 Scientific and Didactic Laboratory of Nanotechnology and Material Technologies, Silesian University of Technology, Towarowa 7a St., 44-100 Gliwice, Poland; akdeshmukh9@gmail.com; 4 Łukasiewicz Research Network, Institute of Non-Ferrous Metals, Sowińskiego 5, 44-100 Gliwice, Poland; tymon.warski@imn.lukasiewicz.gov.pl (T.W.); lukasz.hawelek@imn.lukasiewicz.gov.pl (Ł.H.)

**Keywords:** ZrCu-based alloys, rapid solidification, thermodynamic calculations, structural characterization, thermal analysis, corrosion resistance, tribological tests

## Abstract

Zirconium-based alloys are highly regarded by the research community for their exceptional corrosion resistance, thermal stability, and mechanical properties. In our work, we investigated two newly developed alloys, Zr_42.42_Cu_41.18_Al_9.35_Ag_7.05_ and Zr_46.81_Cu_35.44_Al_10.09_Ag_7.66_, in the form of ingots and ribbons. In the course of our investigation, we conducted a comprehensive structural and thermal analysis. In addition, an examination of the corrosion activity encompassing electrochemical studies and an analysis of the corrosion mechanisms was carried out. To further evaluate the performance of the materials, tests of their mechanical properties were performed, including microhardness and resistance to abrasive wear. Structural analysis showed that both alloys studied had a multiphase, crystalline structure with intermetallic phases. The samples in the form of ribbons showed improved corrosion resistance compared to that of the ingots. The ingot containing a higher content of copper Zr_42.42_Cu_41.18_Al_9.35_Ag_7.05_ was characterized by better corrosion resistance, while showing lower average hardness and a higher degree of abrasive wear based on SEM observations after pin-on-disc tests.

## 1. Introduction

Zirconium-based alloys are important for future technologies due to their spectrum of properties, owing to which they are used in various applications, from structural elements for nuclear reactors [1] to materials for biomedical devices [2,3]. In recent years, many works have been published on ZrCu-based alloys, due to their favorable combination of high glass-forming ability (GFA) [4,5,6], wide supercooled liquid region (SCLR) [6], high thermal stability [5], and excellent mechanical properties [7,8,9,10]. Additionally, ZrCu-based alloys exhibit anticorrosion behavior [5,8,9,10], making them a promising material for various applications, including machinery [5], microelectronics [5,8,9], aerospace [5], and sports equipment [9]. Furthermore, the cost of synthesis of Zr-based alloys is relatively low compared to other materials [11], making them economically feasible for large-scale applications. Zr-based BMGs are a potential material for biomedical applications due to their excellent mechanical strength (∼2 GPa) [2,8,10], low Young’s modulus (80–100 GPa) [2,10], higher elastic limit (2%) [2,8,10], and good wear [2,10] and corrosion resistance [2,10] in a biological environment. The corrosion resistance and mechanical strength of ZrCu-based alloys are largely dependent on the alloying elements. In addition, Al and Ag have been used as alloying elements in the design of new alloys and coatings for antibacterial devices such as hospital surfaces, surgical instruments, and implants [12,13]. Aluminum can also be used in a formation of intermetallic phases with controlled size, morphology, and distribution to achieve favorable functional or structural properties [14].

Zirconium, or more precisely ZrO_2_, is used in dentistry and is considered one of the most aesthetic, biocompatible, and friendly-to-oral-tissue dental materials [15]. The presence of zirconium in the body does not cause allergic reactions. One of its important advantages is its high mechanical strength, which makes it resistant to fractures and other damage. It is also resistant to abrasion. Moreover, Zr is corrosion resistant [16]. It is not affected by nitric and hydrochloric acids and does not dissolve in alkalis. Zirconium has very low thermal conductivity and is difficult to melt.

Copper is one of the most vital elements in the human body and is required for free antioxidant defense, wound healing, and functioning of the immune system [17]. Its complexes have good antitumor potential [17]. Copper also has a strong antibacterial effect. Together with iron, copper enables the body to create red blood cells. Cu plays a role in maintaining healthy bones, blood vessels, nerves, and immune function. In addition, it contributes to the absorption of iron. Sufficient levels of copper in the body can also help prevent cardiovascular disease and osteoporosis. The addition of Cu improves corrosion resistance and tensile strength.

Silver exhibits a multitude of properties that have been successfully employed in the field of medicine. Colloidal silver is a pharmaceutical raw material that is used primarily in the production of prescription drugs. It is a combination of silver, protein, and gelatin. Silver is a natural antimicrobial, thus helping fight against infection and prevent respiratory infections [18]. The daily absorption of silver by humans is estimated to be between 1.8 and 80 μg. Silver is distinguished by its excellent corrosion resistance in a multitude of aqueous solutions and its favorable biocompatibility in vivo. The corrosion behavior of some Zr–Ag alloys in artificial saliva was investigated by Rosalbino et al. [19]. The authors verified that the addition of Ag diminished the susceptibility of Zr to corrosion. The degradation potential of the passive layer of all the tested Zr-based alloys can be considered safe, thereby rendering them potential materials for dental implants. Electrochemical impedance spectroscopy (EIS) measurements indicated that oxide layers formed on the surface of Zr–Ag alloys exhibited superior barrier properties compared to those of the pure Zr alloy. The addition of silver not only promotes the formation of a stable passive oxide layer but also increases its compactness, thereby significantly contributing to the improvement of the corrosion resistance of the studied Zr–Ag alloys. Based on the research results, Bosetti et al. [20] stated that silver has no toxic effect on human cells (i.e., lymphocytes, fibroblasts, and osteoblasts).

The presence of aluminum is associated with an increased risk of developing Alzheimer’s disease, Parkinson’s disease, multiple sclerosis, and amyotrophic lateral sclerosis [21,22]. Furthermore, aluminum ions have been linked to brain damage and disrupted cholesterol metabolism. However, aluminum compounds are employed in the medical field. For example, aluminum hydroxide is utilized to treat hyperacidity and gastric ulcers, while aluminum sulfate is employed to halt bleeding. Al is distinguished by its low weight and high resistance to mechanical loads. Additionally, aluminum exhibits excellent corrosion resistance. The physiological concentration of aluminum in human plasma is 1–2 μg/L. This quantity (or less) indicates a balance between absorption from natural sources and excretion. In a related study, Jiang et al. [23], investigated the mechanical properties of ZrAl binary alloys. It was observed that as the Al content in the Zr-based alloy increased, the strength increased and the elongation decreased. The addition of 9% at. Al resulted in the achievement of optimal mechanical properties, exhibiting a strength of 1122 MPa and an elongation of 9.6%. The introduction of the article [24] demonstrates that the addition of a minor content of aluminum to Zr–Cu alloy has the effect of enhancing compressive plasticity, which is attributed to the formation and branching of the shear bands. Roman et al. [25] analyzed FeMnSi–Al alloy with a new chemical composition as a potential biodegradable metallic material. The authors reported that more research on alloy cytotoxicity must be performed because of the possibility of different diseases.

In light of the aforementioned considerations, the present work entails the design of novel compositions of Zr_42.42_Cu_41.18_Al_9.35_Ag_7.05_ and Zr_46.81_Cu_35.44_Al_10.09_Ag_7.66_ alloys. The objective is to achieve favorable structure, along with exemplary corrosion resistance and mechanical properties, thereby positioning these compositions as promising candidates for antibacterial biomedical devices. The article analyzes the impact of rapid solidification (melt-spinning) on the structure and compares the proposed chemical compositions in terms of corrosion resistance, hardness and abrasive wear resistance.

## 2. Materials and Methods

### 2.1. Preparation of the Ingots and Ribbons

Two different master alloys (ingots) of compositions of Zr_42.42_Cu_41.18_Al_9.35_Ag_7.05_ and Zr_46.81_Cu_35.44_Al_10.09_Ag_7.66_ were prepared using high purity elements (99.9%) of Zr, Cu, Al, and Ag. These ingots were synthesized by induction melting using an NG-40 induction generator (Łukasiewicz Research Network, Gliwice, Poland) in a ceramic crucible, under an argon atmosphere with technical purity (99.9%). The master alloys were remelted several times for process repeatability analysis. No weight loss was observed during the melting process. The master alloys were cast in the form of ribbons using the melt-spinning method, using the Bühler Melt Spinner SC station (Edmund Bühler GmbH, Hechingen, Germany) with a surface speed of 30 m/s using argon as protective atmosphere. The casting temperature of ribbons was about 1400 °C. The measured thickness and width of the ribbons were found to be 30 μm and 5 to 6 mm, respectively. The ejection pressure of 0.3 bar and a gap of 2 mm from the quartz nozzle to the wheel gap were fixed.

### 2.2. Structural Investigations and Thermal Analysis

X-ray diffraction (XRD) analysis was performed for phase identification in the ingots and rapidly cooled ribbons. It was carried out using a Rigaku Mini Flex 600 (Rigaku, Tokyo, Japan), equipped with a copper tube as an X-ray radiation source (Cu-Kα, λ = 1.54 Å) and a D/TEX strip detector. The diffraction patterns were recorded with varying Bragg’s angles from 10° to 90° with step size of 0.02°. The microstructures of Zr_42.42_Cu_41.18_Al_9.35_Ag_7.05_ and Zr_46.81_Cu_35.44_Al_10.09_Ag_7.66_ ingots were analyzed using a scanning electron microscope (Phenom ProX, Thermo Fisher Scientific, Waltham, MA, USA). The chemical element maps were performed using EDX. Differential scanning calorimetry (DTA) analysis of ribbons was provided to determine the crystallization mechanism using a NETSCH Jupiter STA 449 F3 thermal analyser (Netsch, Selb, Germany). The DTA curves were recorded at 10 °C·min^−1^ for heating and cooling under a protective atmosphere of argon.

### 2.3. Corrosion Studies

Electrochemical measurements were performed to investigate the corrosion resistance of the samples studied, both in the form of ingots and ribbons. These measurements were carried out at a temperature that simulates the human body (37 °C) in Ringer solution (8.6 g·dm^−3^ NaCl, 0.3 g·dm^−3^ KCl, and 0.48 g·dm^−3^ CaCl_2_·6H_2_O) using an Autolab 302 N potentiostat (Metrohm AG, Herisau, Switzerland). An assembly for potentiostat consists of the saturated calomel electrode (reference electrode) and the counter platinum electrode. All samples were tested with 3600 s of open circuit potential (*E_OCP_*). Potentiodynamic curves with Tafel’s extrapolation were recorded in a potential range from *E_OCP_* − 250 mV to *E_OCP_* + 250 mV with a scan rate of 1 mV·s^−1^. Furthermore, the corrosion potential (*E_corr_*) and the corrosion current density (*j_corr_*) were calculated by Tafel’s extrapolation using the cathodic and anodic branches of the polarization curves. After electrochemical measurements in Ringer’s solution, the surfaces of the corroded samples with corrosion products were observed using a scanning electron microscope (SEM) (Phenom ProX, Thermo Fisher Scientific, Waltham, MA, USA).

### 2.4. Hardness and Tribological Measurements

Hardness tests on the ZrCu-based alloys in the as-prepared state were performed using a Future Tech FM-700 Vickers (Future Tech, Tokyo, Japan) hardness testing instrument with a load of 100 g for 15 s. Tribological tests for as-cast samples were performed using the pin-on-disc method with CSM Instruments (Peseux, Switzerland). The radius of the wear track was 1.5 mm. A counter-sample was a ball made of 100Cr6 steel (*d* = 6 mm). The linear speed was 0.01 m·s^−1^, and a load of 10 N was applied. The measuring time for every sample was 1 h. Observations of the wear tracks were carried out using a scanning electron microscope (Phenom ProX, Thermo Fisher Scientific, Waltham, MA, USA).

## 3. Results and Discussion

### 3.1. Structural Analysis

**Figure 1:**
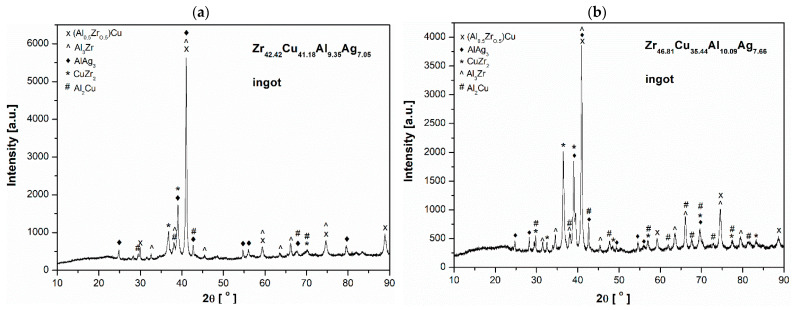
X-ray diffraction patterns of ingots of the Zr_42.42_Cu_41.18_Al_9.35_Ag_7.05_ (**a**) and Zr_46.81_Cu_35.44_Al_10.09_Ag_7.66_ (**b**) alloys.

**Figure 2:**
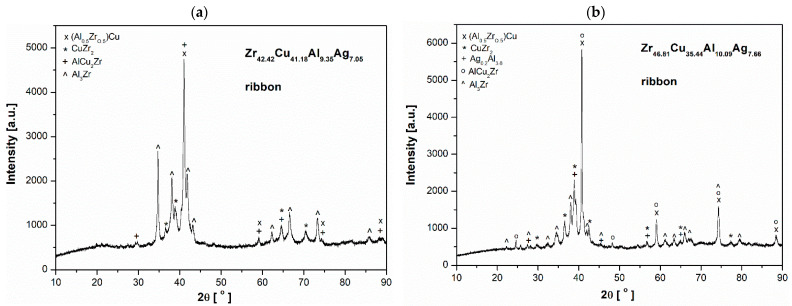
X-ray diffraction patterns of ribbons of the Zr_42.42_Cu_41.18_Al_9.35_Ag_7.05_ (**a**) and Zr_46.81_Cu_35.44_Al_10.09_Ag_7.66_ (**b**) alloys.

The samples studied of Zr_46.81_Cu_35.44_Al_10.09_Ag_7.66_ and Zr_42.42_Cu_41.18_Al_9.35_Ag_7.05_ alloys, both in the form of ingots and ribbons, had a crystalline structure. The X-ray diffraction patterns for them are presented in Figure 1 and Figure 2. In the alloy ingots of both studied alloys the characteristic peaks of (Al_0.5_Zr_0.5_)Cu, Al_3_Zr, AlAg_3_, CuZr_2_, and Al_2_Cu were identified. The ribbon samples exhibited the following phases: (Al_0.5_Zr_0.5_)Cu, CuZr_2_, and Al_3_Zr. Furthermore, the Al_0.2_Ag_3.8_ phase was identified in the alloy with a higher Zr content. Despite the sharp crystalline peaks in both rapidly quenched alloys, noticeable broad diffraction lines between 35 and 45° can be observed that confirm the formation of the glassy matrix. This range is aligned with the glassy alloys [26,27,28].

**Figure 3:**
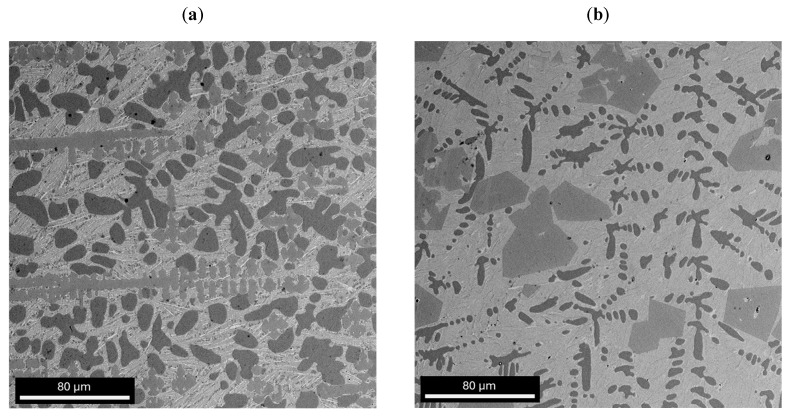
SEM images of (**a**) Zr_42.42_Cu_41.18_Al_9.35_Ag_7.05_, and (**b**) Zr_46.81_Cu_35.44_Al_10.09_Ag_7.66_ alloys.

**Figure 4:**
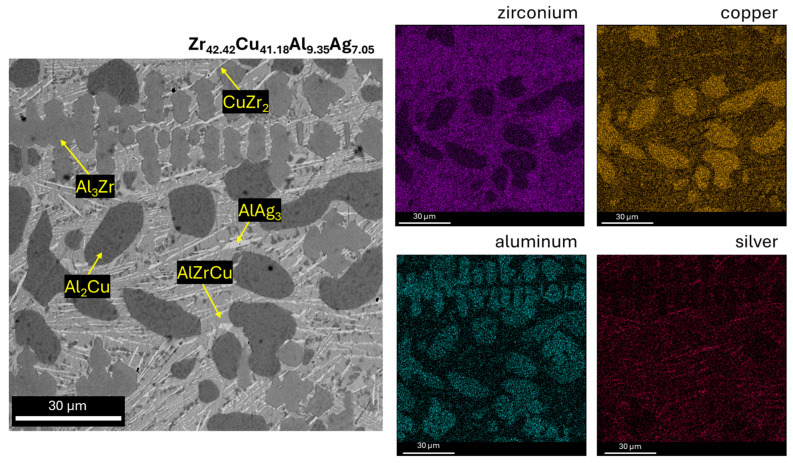
SEM image of as-cast Zr_42.42_Cu_41.18_Al_9.35_Ag_7.05_ alloy with EDX maps.

**Figure 5:**
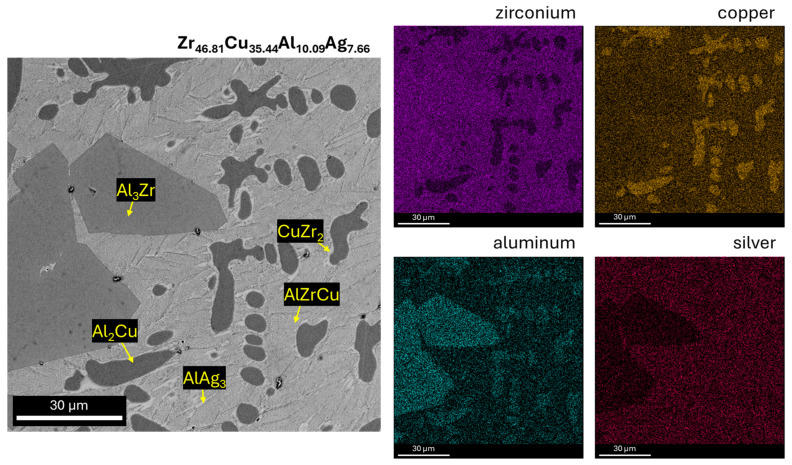
SEM image of as-cast Zr_46.81_Cu_35.44_Al_10.09_Ag_7.66_ alloy with EDX maps.

Figure 3, Figure 4 and Figure 5 show SEM observations of the alloys studied. The presence of identified phases was confirmed by EDX analysis (Figure 4 and Figure 5). The structure of the Zr_42.42_Cu_41.18_Al_9.35_Ag_7.05_ alloy was characterized by presence of an AlZrCu phase matrix, on which very fine plates of the AlAg_3_ phase (which appear when the heating temperature is increased to 450 °C [29]) were visible. The Al_3_Zr phase was identified in the form of a dendritic structure. The globular precipitates confirm the presence of the Al_2_Cu phase due to the high aluminum and copper content in the EDX maps. The Al_2_Cu phase was surrounded at the grain boundaries by very thin amounts of CuZr_2_, which is a stable phase with a body-centered tetragonal crystal structure with six atoms per unit of cell [30]. In the work [31], it was reported that the CuZr_2_ phase should have two precipitating forms. First, when a decrease in the solubility of Zr and Cu in the solid state with a decrease in temperature is observed, and the second—the eutectoid CuZr_2_ phase—when the temperature is lower than 820 °C. The Zr_46.81_Cu_35.44_Al_10.09_Ag_7.66_ alloy was also composed of AlZrCu-phase matrix; however, the amount of AlAg_3_ phase in the form of plates was lower. The Al_3_Zr phase was identified for the as-cast alloy with a higher zirconium content in the form of sharp and equal shapes. It should be mentioned that the presence of other alloying elements, such as Zr and Cu, may influence the precipitation of Al_3_Zr, causing the different precipitate morphologies of this phase [32,33]. Al_2_Cu was also observed for this alloy with similar shapes, however, the growth of this dendritic phase was visible in the Zr_42.42_Cu_41.18_Al_9.35_Ag_7.05_ alloy. However, the Al_2_Cu phase was also surrounded at grain boundaries by the CuZr_2_ phase, in a lower content compared to the Zr_42.42_Cu_41.18_Al_9.35_Ag_7.05_ alloy. Moreover, the chemical composition recorded during EDX analysis from the areas of Figure 4 and Figure 5 is presented in Table 1.

### 3.2. Thermal Analysis and Glass-Forming Ability

**Figure 6:**
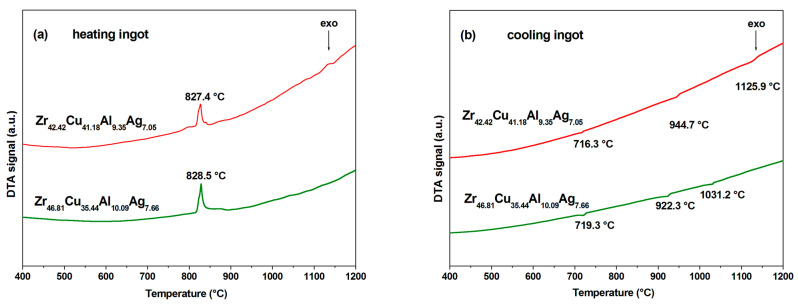
DTA curves of Zr_42.42_Cu_41.18_Al_9.35_Ag_7.05_ and Zr_46.81_Cu_35.44_Al_10.09_Ag_7.66_ ingots recorded after heating (**a**) and cooling (**b**).

Figure 6 presents differential thermal analysis (DTA) curves for the ingots Zr_42.42_Cu_41.18_Al_9.35_Ag_7.05_ and Zr_46.81_Cu_35.44_Al_10.09_Ag_7.66_ recorded after heating and cooling processes. The results of the analysis showed that the DTA curves for both studied alloy ingots have a similar shape. During the heating process, the temperature of the ingots increased evenly. Exothermic effects related to heat release occurred at similar temperatures, reaching an extreme point at temperatures of 827.4 and 828.5 °C for the Zr_42.42_Cu_41.18_Al_9.35_Ag_7.05_ and Zr_46.81_Cu_35.44_Al_10.09_Ag_7.66_ alloy ingots, respectively (Figure 6a). These temperatures are probably associated with the formation of CuZr_2_ phase in the AlZrCu phase matrix [31].

However, during the cooling process, three small endothermic reactions occurred at temperatures of 716.3, 944.7, and 1125.9 °C for the Zr_42.42_Cu_41.18_Al_9.35_Ag_7.05_ and 719.3, 922.3, and 1031.2 °C for the Zr_46.81_Cu_35.44_Al_10.09_Ag_7.66_ alloy. It can be seen that the last two reaction temperatures related to heat absorption for the alloy with a higher Zr content are lower than the temperatures for the second alloy (Figure 6b).

Zhang et al. [34] analyzed the DTA curves of Zr-based alloys with a heating rate of 20 K min^−1^. In the case of the Zr_41_Ti_14_Cu_12_Ni_10_Be_23_ BMG, the crystallization exothermic peak occurred subsequent to the large endothermic peak, which corresponded to the melting process. The addition of magnesium and yttrium to Zr BMG resulted in notable alterations to the crystallization and melting processes. The DTA curve of the Zr_55_Al_20_Co_20_Cu_5_ alloy prepared by arc melting was examined in work [35]. The researchers observed that the alloy underwent a two-step exothermic solidification process, with T1 occurring at a temperature of 987 °C. In the ZrCuAgNiAl glasses [36], the DTA curves were recorded at 10 K min^−1^. The results obtained indicated that the exothermic effects observed at 840 °C were followed by endothermic reactions in the range from 380 to 460 °C.

The higher negative enthalpies favor the formation of various types of stable polyhedral clusters (icosahedral/decahedral) resulting the frustration during the crystallization of material [10,23]. This advocates the higher negative value of the enthalpy of mixing (${∆H}_{Chem}$) among the constituent elements to form bulk metallic glasses (BMGs) [10,23]. The extended sub-regular solution model for ternary alloy proposed by Gallego et al. [37] and Miedema’s semi-empirical model [38] for binary alloys was used to calculate the ${∆H}_{Chem}$ for the present ternary alloys. Normalized mismatch entropy ($S_{\sigma }/k_{B}$) represents the randomness in the system resulting from the different atomic sizes of the constituent elements that ease the formation of BMGs with high GFA [23]. An empirical relation proposed by Mansoori et al. [39] was used to calculate the ${∆S}_{\sigma }/k_{B}$. Prabhu et al. [9,23] discussed the formation of the covalent bonds due to the overlapping of electron clouds at the higher oxidation states. Owing to this, ${∆S}_{\sigma }/k_{B}$ was calculated based on the covalent radius of the constituent elements. Cu = 1.17 Å, Zr = 1.45 Å, Al = 1.25 Å, Ag = 1.34 Å [23,40]. Being a dimensionless quantity, multiplication of ${∆S}_{\sigma }/k_{B}$ with ${∆H}_{Chem}$, denoted by X, will serve as a yardstick to identify the glass-forming compositions (GFCs) with higher disorder and negative enthalpy of mixing [41]. Detailed formulation of ${∆H}_{Chem}$ and ${∆S}_{\sigma }/k_{B}$ can be found in our previous article [42]. This is in line with the empirical rules proposed by Inoue et al. [43] for the synthesis of BMGs. The present quaternary compositions Cu_41.18_Zr_42.42_Al_9.35_Ag_7.05_ (−4.01 kJ/mol) and Cu_35.45_Zr_46.81_Al_10.09_Ag_7.65_ (−3.84 kJ/mol) are close to the reported compositions Cu_42_Zr_42_Al_8_Ag_8_ [44] (−3.95 kJ/mol) and Cu_36_Zr_48_Al_8_Ag_8_ [44] (−3.84 kJ/mol), respectively, which form the complete glassy structure. It can be seen that the value of parameter X for Cu_41.18_Zr_42.42_Al_9.35_Ag_7.05_ is higher compared to the reported glassy alloy. On the other hand, value of parameter X for Cu_35.45_Zr_46.81_Al_10.09_Ag_7.65_ is identical to the reported composition. Parameter X ranges from −3.46 kJ/mol to −5.11 kJ/mol. Jiang et al. [45] reported the partial glassy structure in Cu_(4.5/5.5)46_Zr_47_Al_7_Ag_(1/5.5)46_, Cu_(5/6)46_Zr_47_Al_7_Ag_(1/6)46_, Cu_(6/7)46_Zr_47_Al_7_Ag_(1/7)46_, Cu_(4/5)48_Zr_45_Al_7_Ag_(1/5)48_, Cu_(4/5)42_Zr_50_Al_8_Ag_(1/5)42_, Cu_(5/6)42_Zr_50_Al_8_Ag_(1/6)42_, Cu_(3/4)44_Zr_48_Al_8_Ag_(1/4)44_, Cu_(7/8)44_Zr_48_Al_8_Ag_(1/8)44_, Cu_(5/6)46_Zr_46_Al_8_Ag_(1/6)46_, Cu_(5/6)38_Zr_53_Al_9_Ag_(1/6)38_, Cu_(4.5/5.5)40_Zr_51_Al_9_Ag_(1/5.5)40_, and Cu_(5/6)42_Zr_49_Al_9_Ag_(1/6)42_. Value of parameter X for these alloys is −3.87 kJ/mol, −3.97 kJ/mol, −4.13 kJ/mol, −3.76 kJ/mol, −3.71 kJ/mol, −3.91 kJ/mol, −3.46 kJ/mol, −4.21 kJ/mol, −3.94 kJ/mol, −3.81 kJ/mol, −3.78 kJ/mol, and −3.91 kJ/mol, respectively. This indicates that glassy phase in the Cu–Zr–Al–Ag alloy system is composition sensitive. This indicates that both Cu and Ag strongly influence the GFA in the Cu–Zr–Al–Ag alloy. This can be evidenced from the XRD pattern, as illustrated in Figure 2. For the sample in the form of a ribbon of Zr_42.42_Cu_41.18_Al_9.35_Ag_7.05_, the XRD intensity is lower compared to the same sample form of Zr_46.81_Cu_35.44_Al_10.09_Ag_7.66_. Similarly, the ribbon sample of Zr_42.42_Cu_41.18_Al_9.35_Ag_7.05_ exhibits broader diffraction hump compared to the ribbon sample Zr_46.81_Cu_35.44_Al_10.09_Ag_7.66_, as illustrated in Figure 2a and Figure 2b, respectively.

### 3.3. Corrosion Behavior

**Figure 7:**
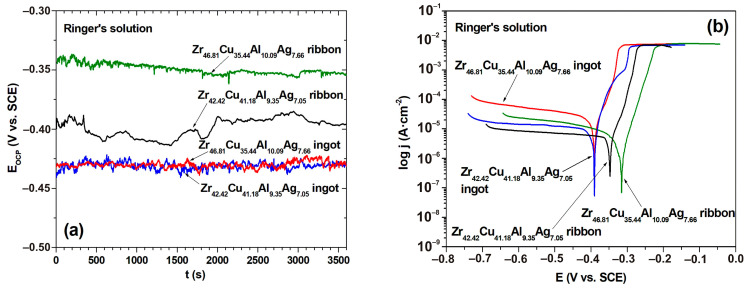
Changes in the open-circuit potential with time (**a**), and polarization curves (**b**) in Ringer’s solution at 37 °C for samples in the form of ingots and ribbons of Zr-based alloys.

In addition to mechanical properties and biocompatibility, investigation of the biocorrosion behavior of the alloy is crucial for biomedicine application [46]. Therefore, the corrosion resistance performance of the samples in the form of ingots and ribbons was investigated by varying the *E_OCP_* with time in Ringer’s solution at a temperature of 37 °C for 3600 s (Figure 7a). The *E_OCP_* potentials for both ingots of Zr_42.42_Cu_41.18_Al_9.35_Ag_7.05_ and of Zr_46.81_Cu_35.44_Al_10.09_Ag_7.66_ were found to be stationary around 0.43 V over the complete duration of measurements. Both ribbon samples of Zr-based alloys showed higher *E_OCP_* compared to the ingots studied. In particular, the ribbon sample of the Zr_46.81_Cu_35.44_Al_10.09_Ag_7.66_ alloy showed better corrosion resistance compared to the ribbon of the second alloy moving in the positive direction (*E_OCP_* for the ribbons of Zr_46.81_Cu_35.44_Al_10.09_Ag_7.66_ and Zr_42.42_Cu_41.18_Al_9.35_Ag_7.05_ were −0.353 and −0.396 V, respectively). Furthermore, the *E_OCP_* curve for the ribbon sample of Zr_46.81_Cu_35.44_Al_10.09_Ag_7.66_ was stabilized.

Figure 7b shows the potentiodynamic polarization curves for all of the studied samples in Ringer’s solution at 37 °C. The basic electrochemical parameters obtained by Tafel’s extrapolation of the polarization curves are presented in Table 2. The corrosion potential for both ingots was found to be identical around 0.39 V. The slightly higher corrosion resistance of the Zr_42.42_Cu_41.18_Al_9.35_Ag_7.05_ alloy may be attributed to its higher copper content (41.18 at.% compared with 35.44 at.% content for the second alloy studied) [47]. Significant curve change was observed for the ribbon samples of the alloys Zr_42.42_Cu_41.18_Al_9.35_Ag_7.05_ and Zr_46.81_Cu_35.44_Al_10.09_Ag_7.66_, as illustrated in Figure 7b. Furthermore, the curves for the Zr-based ribbons were located in a lower current range (around 10^−5^ A·cm^−2^) than ingot samples of the same material (about 10^−4^ A·cm^−2^). The corrosion potentials for the ribbons of Zr_46.81_Cu_35.44_Al_10.09_Ag_7.66_ and Zr_42.42_Cu_41.18_Al_9.35_Ag_7.05_ were −0.317 and −0.350 V, respectively. The better corrosion resistance of the ribbon sample of the alloy with a higher Ag content was confirmed by the results for polarization resistance (*R_p_*). The value of *R_p_* for this alloy was 5.31 kΩ·cm^2^, and it was higher in comparison to the second ribbon sample (*R_p_* was 2.18 kΩ·cm^2^). This suggests better corrosion resistance for the ribbon of the Zr_46.81_Cu_35.44_Al_10.09_Ag_7.66_ alloy.

Rosalbino et al. [19] studied the corrosion behavior of Zr–Ag alloys (with a 1, 3, and 5 wt.% Ag content) in artificial saliva to evaluate their potential use as dental materials. The test results showed that the addition of silver alloy reduced the susceptibility of zircon to corrosion, as illustrated by the shift of the *E_OCP_* and *E_corr_* potentials to more positive values compared to pure Zr [19]. The performance of glassy ribbons is better compared to the Zr_46_(Cu_4.5/5.5_Ag_1/5.5_)_46_Al_8_ BMG reported by Sun et al. [46] for biomedical applications. The corrosion potential for Zr_46_(Cu_4.5/5.5_Ag_1/5.5_)_46_Al_8_ BMG was −0.388 ± 0.04 V and −0.555 ± 0.03 V in 0.9% NaCl and Hank’s solutions, respectively [46]. Similarly, present ribbon samples of Zr_46.81_Cu_35.44_Al_10.09_Ag_7.66_ and of Zr_42.42_Cu_41.18_Al_9.35_Ag_7.05_ showed better performance compared to the Zr_46_(Cu_0.82_Ag_0.18_)_46_Al_8_ BMG [48]. The corrosion potential for Zr_46_(Cu_0.82_Ag_0.18_)_46_Al_8_ BMG was −0.376 V.

**Figure 8:**
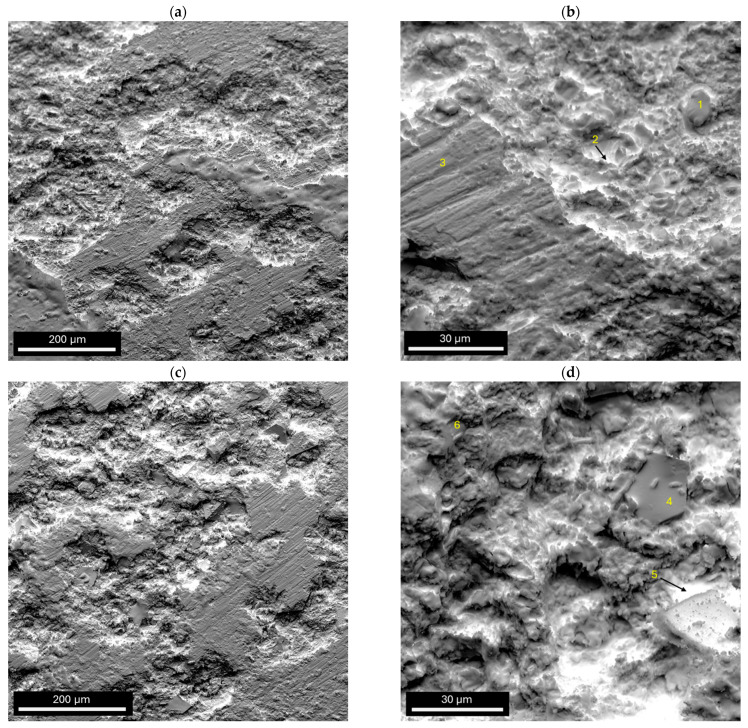
Surface morphology of (**a**,**b**) Zr_42.42_Cu_41.18_Al_9.35_Ag_7.05_ and (**c**,**d**) Zr_46.81_Cu_35.44_Al_10.09_Ag_7.66_ ingots after electrochemical tests in Ringer’s solution at 37 °C.

After electrochemical corrosion tests, the ingot samples with corrosion products were observed using SEM (Figure 8). Furthermore, the EDX analysis of the chemical composition from selected points marked in Figure 8b,d is presented in Table 3. In the EDX analysis, in addition to oxygen, the elements constituting ions in Ringer’s solution (Na, Cl, K and Ca) were also taken into account.

The surface damage of both studied samples was pitting corrosion [49]. No microcracks were visible. It can be stated that the ingot sample of the alloy with a lower Zr content is slightly less damaged compared to the second sample studied. Furthermore, it can be seen that the corrosion products were situated in the center of the pits. Some corrosion products fell off the surface of the ingot sample of the Zr_46.81_Cu_35.44_Al_10.09_Ag_7.66_ alloy (Figure 8d). Mudali et al. in [50] observed that pits spread spherically at a constant rate in all directions. Corrosion products begin to settle in the center of the pit, yet in the central part of the pits, bright oxide products are formed. Furthermore, it can be assumed that galvanic microcells were formed in a corrosive environment in multiphase alloys with an intermetallic phase structure. Kawashima et al. [51] performed SEM and EDX studies after corrosion tests in a 0.5 M NaCl solution for Zr_50_Cu_40_Al_10_ and Al_70_Cu_6_Al_8_Ni_16_ BMGs. The authors [51] demonstrated the appearance of pitting corrosion for the Zr_50_Cu_40_Al_10_ alloy. Inside the pit, copper enrichment and zirconium and aluminum deficiency were identified, which was explained as preferential dissolution of Zr and Al [51]. The authors [51] suggested that the corrosion products that occur inside the pits were CuCl, Cu_2_O, or CuO. In other works [52,53], it was found that copper-rich compounds could cause a galvanic coupling effect enhancing the dissolution of the glassy phase.

It should be mentioned that most zirconium-based alloys located in an oxygen-containing environment have a thin oxide layer (2 to 5 nm) on their surface. ZrO_2_ formed has a protective effect, limiting the access of oxidizing compounds inside the metal. However, the increase in the thickness of the ZrO_2_ layer on the alloy surface depends on the kinetics of O_2_ diffusion through this layer. Many studies indicated that zirconium oxidation occurs through the migration of oxygen ions through the oxide layer, through grain boundaries, or through the mass [54,55].

EDX analysis of the selected points confirmed that the pits contained highly oxidized corrosion products (see points 1–2, 4–6). In this work, it can be observed that, similarly to the literature, there was more copper inside the pits than zirconium and aluminium (see points 1, 2, 5, and 6). This indicates the local dissolution of Zr and Al. Outside the pits, the share of zirconium was larger, indicating the formation of a passive layer (see points 3 and 4). Microscopic observations confirmed the results of the corrosion studies, in which the ingot sample of the Zr_42.42_Cu_41.18_Al_9.35_Ag_7.05_ alloy was slightly more resistant to corrosion than Zr_46.81_Cu_35.44_Al_10.09_Ag_7.66_ (the ingot exhibited E_corr_ of −0.390 V and j_corr_ of 3.52 μA·cm^−2^, in contrast to the E_corr_ of −0.391 V and j_corr_ of 3.81 μA·cm^−2^ observed in the Zr_46.81_Cu_35.44_Al_10.09_Ag_7.66_). It is likely that this phenomenon is due to the higher Cu and lower Zr content of this alloy.

### 3.4. Mechanical Properties

**Figure 9:**
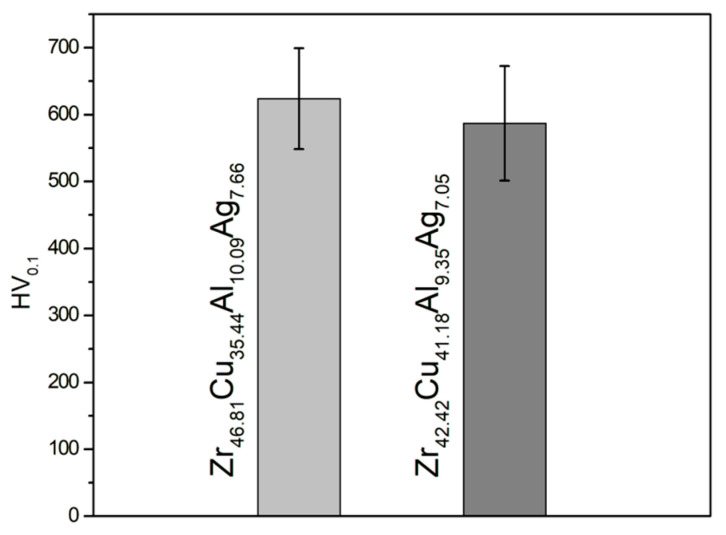
Microhardness of Zr_42.42_Cu_41.18_Al_9.35_Ag_7.05_ and Zr_46.81_Cu_35.44_Al_10.09_Ag_7.66_ ingots.

The results of the average microhardness values of the tested Zr_42.42_Cu_41.18_Al_9.35_Ag_7.05_ and Zr_46.81_Cu_35.44_Al_10.09_Ag_7.66_ alloys in ingot form of ingots are presented in Figure 9. The Zr_46.81_Cu_35.44_Al_10.09_Ag_7.66_ alloy was characterized by an average microhardness of 623.8 (±75.3) HV, while for Zr_42.42_Cu_41.18_Al_9.35_Ag_7.05_ a lower value of 587.0 (±85.7) HV was determined. Wen et al. [56], in their work, examined, among other things, microhardness with a load of 100 g for alloys (Cu_46-x_Zr_47_Al_7_Ag_x_)_100-y_Co_y_ (x = 0, 1, 2, 3, 4 and y = 0, 0.5, 1, 1.5) in the form of rods with a diameter of 3 mm. The Ag_0_–Co_0_ alloy with a crystalline structure, containing B2-CuZr, Cu_10_Zr_7_, and CuZr_2_ phases, was characterized by the lowest microhardness (467.1 HV). As silver increased in the alloys studied by Wen et al. [56], the microhardness increased, but the structure of these alloys was amorphous. The highest microhardness value was 563.2 HV for the Ag_3_–Co_0_ alloy, while above 4 at.% silver a decrease in microhardness (548.9 HV) was recorded. Similar conclusions were reported by Zhang et al. [57], who also found that the Zr_48_Cu_45_Al_7_ alloy doped with silver is characterized by higher microhardness. In this work, the alloy with a higher content of zirconium, aluminum, and silver was characterized by higher hardness. Based on SEM microstructure images and matched reflections in the XRD diffractograms, it can be assumed that the share of the Al_3_Zr phase was higher for the Zr_46.81_Cu_35.44_Al_10.09_Ag_7.66_ alloy. The Al_3_Zr phase is an intermetallic phase with high hardness, as described, among others, by Umeda et al. [58]. The work [55] indicated the microhardness of the Al_3_Zr intermetallic compound equal to 645 HV, therefore it can be assumed that the Al_3_Zr phase contributed to the increase in microhardness in the Zr_42.42_Cu_41.18_Al_9.35_Ag_7.05_ alloy. However, it can be observed that the alloy Zr_46.81_Cu_35.44_Al_10.09_Ag_7.66_ was also characterized by a uniform silver content in the matrix, while in the structure of the Zr_42.42_Cu_41.18_Al_9.35_Ag_7.05_ alloy the AlAg_3_ phase appeared in the form of needle precipitates. Therefore, it can generally be stated that Zr–Cu–Al–Ag alloys with a higher copper content at the expense of zirconium, aluminum, and silver are characterized by lower hardness.

**Figure 10:**
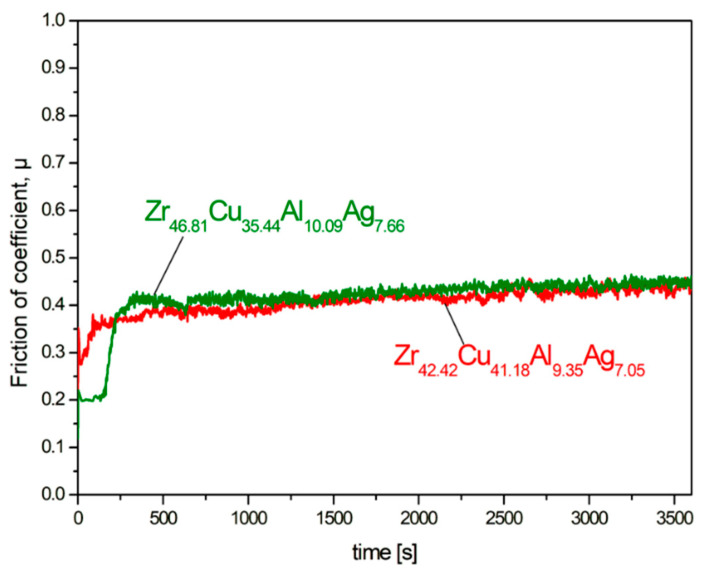
Friction of coefficient in a function of time curves recorded for studied Zr_42.42_Cu_41.18_Al_9.35_Ag_7.05_ and Zr_46.81_Cu_35.44_Al_10.09_Ag_7.66_ ingots during pin-on-disc tests.

**Figure 11:**
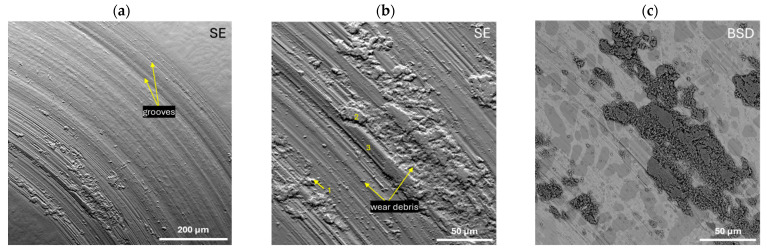
Wear track after the pin-on-disc test for the Zr_42.42_Cu_41.18_Al_9.35_Ag_7.05_ alloy in the SE mode with marked wear mechanisms (**a**,**b**) and in the BSD mode with marked points for EDX analysis (**c**).

**Figure 12:**
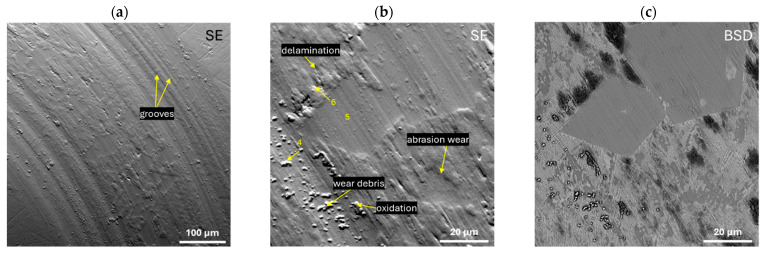
Wear track after the pin-on-disc test for the Zr_46.81_Cu_35.44_Al_10.09_Ag_7.66_ alloy in the SE mode with marked wear mechanisms (**a**,**b**) and in the BSD mode (**c**).

Pin-on-disc measurements were carried out for the studied Zr_42.42_Cu_41.18_Al_9.35_Ag_7.05_ and Zr_46.81_Cu_35.44_Al_10.09_Ag_7.66_ alloys in the form of ingots to assess their resistance to abrasive wear. The curves of the friction coefficient (*μ*) as a function of time (1 h) are presented in Figure 10. Moreover, the EDX analysis of the chemical composition of the selected points marked in Figure 11b and Figure 12b is presented in Table 4. Both alloys were characterised by similar average values of the friction coefficient: 0.41 ± 0.03 for Zr_42.42_Cu_41.18_Al_9.35_Ag_7.05_ and 0.42 ± 0.06 for Zr_46.81_Cu_35.44_Al_10.09_Ag_7.66_. Increasing the friction of the coefficient curves as a function of time at the initial measurement time may suggest the presence of an oxide layer on the surface prior to the test. Figure 11 and Figure 12 show SEM images of wear traces after tribological tests for the alloys Zr_42.42_Cu_41.18_Al_9.35_Ag_7.05_ and Zr_46.81_Cu_35.44_Al_10.09_Ag_7.66_, respectively. For the Zr_42.42_Cu_41.18_Al_9.35_Ag_7.05_ alloy, the appearance of grooves (Figure 11a) and wear debris (Figure 11b) was observed. The SEM image in BSD mode (Figure 11c) together with the EDX point EDX analysis allowed us to conclude that oxidation wear occurred in some areas of the matrix, while the Al_2_Cu and Al_3_Zr phases showed increased wear resistance, because only the wear mechanism was not observed in them. For the alloy Zr_46.81_Cu_35.44_Al_10.09_Ag_7.66_, the occurrence of grooves (Figure 12a), wear debris, delamination, and the abrasive wear mechanism (Figure 12b) was observed. EDX analysis from the selected points confirmed the presence of highly oxidized wear debris (see points 1 and 4) and oxidation due to elevated temperature due to friction during the pin-on-disc test. Zhang et al. [57] found that for bulk metallic glasses Zr_48_Cu_45−x_Al_7_Ag_x_ (x = 0, 2, 5, and 8 at%), a low coefficient of friction and high wear resistance are characteristic for alloys with a higher silver content. Moreover, there is a relationship between greater hardness and higher wear resistance [57]. Although the Zr_42.42_Cu_41.18_Al_9.35_Ag_7.05_ alloy was characterized by a slightly lower friction of coefficient based on the pin-on-disc curves, the SEM images show that the Zr_46.81_Cu_35.44_Al_10.09_Ag_7.66_ alloy was characterized by more uniform wear compared to an alloy with a higher copper content.

## 4. Conclusions

ZrCu-based alloys have the potential for use in biomedical devices and have attracted considerable attention in this regard. The design of biomaterials is dependent on two key factors: corrosion resistance and mechanical properties. In the present study, two alloys of Zr_42.42_Cu_41.18_Al_9.35_Ag_7.05_ and Zr_46.81_Cu_35.44_Al_10.09_Ag_7.66_, in both ingot and ribbon form, were investigated. Based on the research results, the following conclusions were formulated:

1. The samples of the alloys Zr_42.42_Cu_41.18_Al_9.35_Ag_7.05_ and Zr_46.81_Cu_35.44_Al_10.09_Ag_7.66_, both ingot and ribbon forms, exhibited a crystalline structure.
2. In both alloy ingots of ZrCu-based alloys, the characteristic peaks of (Al_0.5_Zr_0.5_)Cu, Al_3_Zr, AlAg_3_, CuZr_2_, and Al_2_Cu were identified. The ribbon samples exhibited the following phases: (Al_0.5_Zr_0.5_)Cu, CuZr_2_, and Al_3_Zr. Furthermore, the A_l0.2_Ag_3.8_ phase was identified in the Zr_46.81_Cu_35.44_Al_10.09_Ag_7.66_ alloy.
3. The DTA curves for the two alloy ingots exhibit a comparable shape. During the heating process, the temperature of the analyzed alloy ingots increased in a uniform manner. Exothermic effects were observed at comparable temperatures, reaching a maximum at 827.4 and 828.5 °C for the alloy ingots Zr_42.42_Cu_41.18_Al_9.35_Ag_7.05_ and Zr_46.81_Cu_35.44_Al_10.09_Ag_7.66_, respectively. These temperatures are likely associated with the formation of the CuZr_2_ phase.
4. Ingot samples of both ZrCu-based alloys were characterized by higher corrosion activity compared to that of the ribbon form. Ribbons with a higher Ag content have a higher corrosion resistance. This is confirmed by both the results of open-circuit potential and polarization measurements. For the Zr_46.81_Cu_35.44_Al_10.09_Ag_7.66_ ribbon the corrosion potential (*E_corr_*) was found to be equal to −0.317 V, the corrosion current density (*j_corr_*) was 1.09 μA·cm^−2^, and polarization resistance (*R_p_*) was 5.31 kΩ·cm^2^.
5. The surface damage observed in both ingot samples after corrosion studies was consistent with pitting corrosion. It was found to be less severe in the Zr_42.42_Cu_41.18_Al_9.35_Ag_7.05_ alloy. Microscopic observations of the corrosion products confirmed the corrosion test results, which indicated that the ingot with a higher Cu and lower Zr content exhibited greater resistance to corrosion.
6. The results of the microhardness tests showed that the alloy Zr_46.81_Cu_35.44_Al_10.09_Ag_7.66_ with a lower Cu content exhibited an average microhardness of 623.8 (±75.3) HV, which is indicative of its mechanical durability. Zr_42.42_Cu_41.18_Al_9.35_Ag_7.05_ exhibited a lower value of 587.0 (±85.7) HV.
7. The results of the abrasive wear resistance tests demonstrated that the ingots of both studied alloys exhibited a comparable average friction coefficient; however, the alloy SEM images showed that Zr_46.81_Cu_35.44_Al_10.09_Ag_7.66_ was characterized by more uniform wear compared to an alloy with a higher copper content.

## Figures and Tables

**Table 1:** Chemical composition from SEM images areas of studied alloys on the basis of EDX analysis.

Alloy	Zr	Cu	Al	Ag
Zr_42.42_Cu_41.18_Al_9.35_Ag_7.05_	44.02	35.49	12.28	8.22
Zr_46.81_Cu_35.44_Al_10.09_Ag_7.66_	46.87	30.71	12.86	9.55

**Table 2:** Polarization results of Zr_42.42_Cu_41.18_Al_9.35_Ag_7.05_ and Zr_46.81_Cu_35.44_Al_10.09_Ag_7.66_ alloys in Ringer’s solution at 37 °C.

Alloy	Sample	*E*_OCP_ [V] (±0.01)	*E*_corr_ [V] (±0.01)	*R*_p_ [kΩ·cm^2^] (±0.1)	*j*_corr_ [μA·cm^−2^] (±0.1)
Zr_42.42_Cu_41.18_Al_9.35_Ag_7.05_	ingot	−0.427	−0.390	1.49	3.52
	ribbon	−0.396	−0.350	2.18	1.79
Zr_46.81_Cu_35.44_Al_10.09_Ag_7.66_	ingot	−0.432	−0.391	0.97	3.81
	ribbon	−0.353	−0.317	5.31	1.09

**Table 3:** Chemical composition from selected points on the basis of EDX analysis of Zr_42.42_Cu_41.18_Al_9.35_Ag_7.05_ (1–3 points, Figure 8b) and Zr_46.81_Cu_35.44_Al_10.09_Ag_7.66_ (4–6 points, Figure 8d) ingots after electrochemical tests in Ringer’s solution at 37 °C.

Point Number	Figure	Zr	Cu	Al	Ag	Na	Cl	K	Ca	O
1	8b	7.45	13.48	6.05	1.93	0.87	2.34	0.08	0.07	67.72
2	8b	8.34	13.41	6.92	2.02	1.71	0.45	-	-	67.15
3	8b	36.77	17.86	16.02	1.96	1.22	-	-	-	26.17
4	8d	34.6	16.49	14.96	2.09	0.95	-	-	-	30.90
5	8d	13.85	58.27	1.62	3.05	0.48	0.67	-	-	22.06
6	8d	25.97	33.00	8.85	2.33	0.59	-	-	-	29.26

**Table 4:** Chemical composition from selected points on the basis of EDX analysis of Zr_42.42_Cu_41.18_Al_9.35_Ag_7.05_ (1–3 points, Figure 11b) and Zr_46.81_Cu_35.44_Al_10.09_Ag_7.66_ (4–6 points, Figure 12b) ingots after the pin-on-disc tests.

Point Number	Figure	Zr	Cu	Al	Ag	O
1	11b	10.14	8.03	2.89	2.12	76.82
2	11b	9.85	10.23	3.06	2.31	74.56
3	11b	35.76	27.60	11.58	1.82	23.24
4	12b	14.06	8.60	3.52	3.01	70.81
5	12b	38.72	19.69	14.56	3.33	23.69
6	12b	32.43	23.32	7.67	7.85	28.73

## Data Availability

The original contributions presented in the study are included in the article, further inquiries can be directed to the corresponding author.
